# Fertility regulation as identity maintenance: Understanding the social aspects of birth control

**DOI:** 10.1177/1359105317726367

**Published:** 2017-09-19

**Authors:** Cicely Marston, Alicia Renedo, Gertrude Nsorma Nyaaba

**Affiliations:** 1 London School of Hygiene & Tropical Medicine, UK; 2 Population Council, Ghana

**Keywords:** family planning, Ghana, identity, sexual behaviour, women’s health

## Abstract

We take a dialogical approach to exploring fertility regulation practices and show how they can maintain or express social identity. We identify three themes in educated Ghanaian women’s accounts of how they navigate conflicting social demands on their identity when trying to regulate fertility: secrecy and silence – hiding contraception use and avoiding talking about it; tolerating uncertainty – such as using unreliable but more socially acceptable contraception; and wanting to be fertile and protecting menses. Family planning programmes that fail to tackle such social-psychological obstacles to regulating fertility will risk reproducing social spaces where women struggle to claim their reproductive rights.

## Introduction

As fertility rates around the world fall, more people are spending more of their lives taking action to avoid unwanted or unplanned pregnancies and births. Women’s individual choices and ability to control their fertility are of major policy interest (Family Planning 2020, 2015), yet few attempts have been made to understand the interconnections between fertility regulation and the complex tactics women must use to preserve and develop their social identities. A nuanced exploration of fertility regulation that takes into account women’s subject positions (i.e. positions made available to women in society via particular practices, cultural discourses and social relationships) and the social identities that develop through women’s experiences and negotiation of such subject positions is vital both in its own right to improve understanding of fertility practices and also to help focus family planning policies and programmes. At present, while many surveys quantify contraceptive and abortion practices, a broader social-psychological understanding of contraception in practice is rare; this is also the case for abortion (Macleod et al., 2016).

In this article, we develop a dialogical approach to understanding contraceptive use and contribute to understanding how social and individual dimensions interact to influence fertility regulation. This approach enables us to study fertility regulation as a behaviour that develops in the inter-subjective space between women and society, rather than as purely individual acts. We consider the case of Ghana, which is pioneering fertility decline in West Africa. Despite this decline, reported levels of contraception are unusually low. For instance, in 2012–2014, Kenya and Ghana recorded similar total fertility rates (Kenya – 3.9, Ghana – 4.2 births per woman), but very different levels of contraceptive prevalence among married women – Kenya at 58 per cent more than double Ghana at 27 per cent (Askew et al., 2017).

The situation in Ghana is particularly interesting because unlike in most settings, the lowest use of modern contraceptives (which in this setting comprise mostly injectables and to a lesser extent, implants, oral contraceptive pills and condoms) is recorded among the wealthiest quintile of women and in urban areas (Askew et al., 2017; Ghana Statistical Service, Ghana Health Service and ICF International, 2015; Ghana Statistical Service, Ghana Health Service and ICF Macro, 2009; ICF Macro, 2010; Machiyama and Cleland, 2014). Modern contraceptive use in the capital, Accra, in 2014 was almost the lowest recorded in the country, second only to Northern Region (Ghana Statistical Service, Ghana Health Service and ICF International, 2015). The highest use of modern contraceptives in Ghana is now among women with only primary education (27%), with lower levels recorded among women with secondary education and higher (24%) (Ghana Statistical Service, Ghana Health Service and ICF International, 2015).

A decline in use of modern methods among educated women has also been recorded in other low-middle-income countries such as India (Basu, 2005). We analyse elsewhere limitations of survey questionnaire design that may help explain the seemingly anomalous survey results (Marston et al., in press; Marston et al., 2016), identifying at least three areas where underreporting is likely: abortion, use of emergency contraceptive pills (ECPs) and use of calendar methods of contraception. Others have also highlighted the need to consider social-psychological dimensions of health-related practices in Ghana (Aikins, 2003) and elsewhere (Campbell et al., 2007; Howarth et al., 2004).

Subject positions made available to women in Ghanaian society are framed by a patriarchal ‘gender contract’ (Kuenyehia, 1995), in which women are expected to be good wives and fulfil motherhood duties. Local religious teachings favour abstinence before marriage (Luginaah et al., 2005). Previous studies have shown how Ghanaian women may have low status (Derose et al., 2002; Mill and Anarfi, 2002) and can fear that childlessness will be stigmatising (Fledderjohann, 2012; Tabong and Adongo, 2013). Hormonal method side effects including effects on fertility are often reported to be a concern (Hindin et al., 2014; Krakowiak-Redd et al., 2011); Sossou (2007) found that women in Ghana do not enjoy full reproductive rights owing to cultural norms (childlessness as deviance) and patriarchal practices (pressures on women to be a ‘good wife’, p. 29).

In this article, we explore how women’s social identities and subject positions affect and are affected by their fertility regulation strategies and how a focus on women’s identities and their socially-situated nature can help explain women’s success or otherwise in regulating their fertility.

## Methods

We conducted a qualitative study in Accra, Ghana, to explore fertility regulation strategies among women who had completed a minimum of secondary education – the group that survey data suggest have the most counterintuitive fertility regulation patterns (low contraception yet also low fertility).

### Approach

We draw on dialogical approaches to social identity (Hermans, 2001; Hermans and Dimaggio, 2007; Renedo, 2014; Renedo and Marston, 2011). In a dialogical approach, identities are conceptualised as dynamically constructed through and against others’ voices, assimilating, contesting and/or negotiating subject positions and social expectations. In this conceptualisation, a person’s identity is not intrinsic and fixed, but evolves in dialogue with and in response to the demands of different voices of other individuals (such as a partner) and collective voices (such as voices of their age group, or a cultural or religious group) (Hermans, 2001). A dialogical approach helps us explore the ways in which fertility regulation practices emerge within and from a social setting, helping illuminate how a woman’s relationships, her experience of particular socio-cultural norms (for instance, about womanhood) and subject positions made available to women in the social context (such as the position of bearer of children) help frame and organise her fertility regulation practices.

### Data collection methods

To maximise variety in responses, we sampled a wide range of women currently or previously in unions aged 18–49 years from different locations in Accra. Unions ranged from boyfriend/girlfriend relationships to marriage. We used snowballing and purposive sampling techniques to recruit participants from personal networks, shopping malls, workplaces and universities. To capture different parts of the reproductive life course, interviewees were recruited in groups representing different parts of the life course: aged 18–24, 25–39 and 40–49 years. We recruited participants through ‘snowball’ methods: we identified the first participants from the personal networks of the local Ghanaian research team and by approaching women directly in public places (e.g. markets, workplaces and universities). We wanted to conduct enough interviews and focus groups to reach ‘data saturation’ where key themes are addressed and new data collection adds little information. We conducted more interviews with women in the key reproductive years where fertility regulation is particularly likely to be happening, that is, 25–39 years.

In individual depth interviews with eight 18–24-year olds, twelve 25–39-year olds and five 40–49-year olds, we discussed women’s reproductive lives and relationships and their views on and use of different fertility regulation strategies. We also conducted one focus group discussion with around eight participants for each of the age groups listed (three in total). The groups discussed fertility and contraceptive use, along with different methods for regulating fertility.

Most interviews were conducted by Ghanaian research interviewers in locations convenient for participants, for example, at their homes, workplace or university. A.R., C.M. and G.N.N. conducted one interview each and helped facilitate the focus groups. Interviews lasted approximately 1 hour. Following local advice, participants were given a small fixed payment to cover any costs of participation, for example, travel.

All interviews were audio-recorded, conducted in English or, in some places, Twi – a local language. Interviews were transcribed by Ghanaian transcribers. Although interviewees were welcome to speak in Twi, it was used only in brief phrases such as exclamations and was translated both verbatim and into colloquial English by bilingual speakers within the transcript. Field notes were used to record data on locations, body language and other observations. Transcripts were reviewed and corrected by the research team to ensure verbatim transcription.

All participants received written information about the study and provided informed consent to participate. We received ethical approval from Ghana Health Service Ethical Review Committee and from the (London School of Hygiene and & Tropical Medicine) Research Ethics Committee.

### Analysis

We used a combined inductive thematic and dialogical approach to analysis, in which increasingly abstract themes emerging from the data were identified in a process of ‘constant comparison’. Our coding frame reflected our a priori interest in fertility regulation and was also developed inductively from the entire data set through open coding using line-by-line coding and process-orientated codes (Charmaz, 2006; Saldana, 2009). Themes that emerged were reviewed and refined via repeated rounds of coding and analytic ‘memo-writing’ (Charmaz, 2006). We paid close attention to specific elements of the narrative (e.g. reported speech, appeals to common sense and use of metaphor). Informed by Bakhtin (1981) on dialogue, we also focused on how participants articulated their accounts in a relational way: that is, through and against different voices, addressing and responding to those voices (e.g. my partner, my religious community and other women). During analysis, we engaged in constant reflexive dialogue with one another at intensive analytical workshops (G.N.N. visited the United Kingdom from Ghana to work with C.M. and A.R. on the analysis) and through sharing analytical memos to ensure that our analysis was robust. During these workshops, we also reflected on how interviewees might have participated in the co-production of interview and focus group data. Most interviews were conducted by educated Ghanaian women and so are likely to contain similar tacit understandings and shared meanings that are also constructed in their everyday social lives. Analysis was carried out by Ghanaian (G.N.N.) and non-Ghanaian researchers (C.M. and A.R.), helping us challenge and unpack tacit understandings on both sides.

## Findings

Participants’ accounts of their fertility regulation practices reflected their attempts to reconcile tensions between wanting to exercise autonomy over reproductive choices by delaying childbearing and simultaneously needing to conform to prevailing social norms and expectations that often appeared to work in opposition, such as expectations that women should marry, bear children and conform to partner’s/husband’s desires with respect to reproductive and contraceptive choices. The women navigated these choices using various tactics, including the following.

### Secrecy and silence: ‘your husband will not even know’

Secrecy and silence were key themes in women’s narratives about controlling their fertility. Women accounted for their fertility regulation practices in dialogue with what they expressed as men’s expectations of them. For instance, they narrated discussing using withdrawal as a method of contraception as difficult or impossible in the context of men’s desire for pregnancy and the constraints of the subject positions available to women, where potential to bear children is a crucial characteristic of femininity/womanhood:

I mean he has married you, why would you… because of pregnancy you know? Why would you think that at the, at the tail end of having sex, he should pull out because you will get pregnant. So I think that one and even with where I am coming from, you can’t even mention it [withdrawal] to your husband […]. You can’t tell him that ‘I don’t want to get pregnant so when you are about to come out pull out’: no. Interviewee P (35-years old)

Women accounted for fertility regulation practices and the pros and cons of different methods through and against the narrated voice of male partners and their preferences. From their narratives, women’s default assumption seemed to be that men would make the final decision about contraception and fertility:

Interviewer: So how would you protect yourself from getting pregnant?

Respondent: I don’t know. I pray that he agrees that [to use her preferred method]. If not, we have to give birth to about twenty kids. Interviewee A (30-years old)

Nevertheless, women described silence and secrecy as tactics they could use to try to gain some agency and simultaneously avoid threats to the relationship such as arguments or even abandonment that might be triggered by women’s infractions with respect to the ‘gender contract’, including trying to avoid pregnancy:

R: Some, some of them [contraceptive methods] are very inconvenient, but this one, once you have your jab [i.e. injectable contraceptive], even your husband will not even know or your partner will not even know that you have done something so…[…]

R: That was what I was about say: that, your partner might not even know you are doing it ‘cause you’re… you’re going for it at the facility and he will not go with you […] so…

I: But why don’t people […] tell their partners, or have that discussion?

R1: …why do you think

R2: ‘cause some…

R: …some don’t really agree to birth [control] methods, yeah. They don’t.

R: So they’ll just leave work, go and take the jab and then go back to the workplace. Focus group (25–49-year olds)

Participants often talked about their partners’ concerns about modern hormonal methods affecting women’s fertility and about managing these concerns. For the focus group participants in the quote above, ‘convenience’ may be related to some extent to being able to obtain a contraceptive method during the working day, but the discussion focuses on being able to regulate fertility secretly while giving the appearance of going along with men’s opposition to contraception.

Participants said men objected most strongly to the contraceptive methods that the women themselves trusted the most. Some women said they complied with their partners’ desires with respect to contraceptive methods and reproduction, for instance, by not taking up the method or by abandoning it. Others, however, said they pretended to comply but used ‘forbidden’ methods secretly:

R: Because the condom I didn’t use it often, so I decided to use the Secure [daily oral pill].

I: So why did you start using the Secure?

R: Because my partner doesn’t always agree to use the condom, and it [the condom] burst at times that’s why I was using the Secure.

I: Was he aware you were using the Secure?

R: No, no, he wasn’t aware.

I: Why didn’t you tell him?

R: (laughs) No, no, he wouldn’t agree, because he was saying that there was some side effects so…. Interviewee E (31-years old)

Interviewee E conveys the plural and relational nature of the constraints participants face when trying to meet their reproductive needs (her partner ‘doesn’t always agree’ to use condoms, he ‘wouldn’t agree’ to use pills). Interviewee E uses daily oral contraceptive pills and circumvents her partner’s stated (or anticipated) opposition without his knowledge. Her fertility regulation strategies are more than a simple ‘choice’ to use pills to avoid pregnancy: she is also responding to her own and her partner’s irregular use of condoms and her understandings of her partner’s views on side effects.

One focus group participant told us about a woman who invoked religion to deflect her husband’s questions about why she had not become pregnant:

R1: I know one nurse she’s having only a child, one child. But the husband wants her to have more and she… she wants some space between the children so she says ‘no’. But her husband doesn’t know she is having her family planning method. She is… ah… she is working at the family planning unit […] so she is doing it herself and when she goes home and the husband asks – ‘Eii, this child [speaks in twi] deɛ[here] the space [between pregnancies] is… is getting too much’. She says: ‘Oh, [speaks in Twi] children deɛ [Translation – for children] it’s God who gives’. [Laughter from room]. ‘At the right time, you have another one, but for now if God hasn’t given me any child, I can’t conceive’. So… so that’s what she’s telling the husband. Meanwhile she is having a family planning method right at her facility:

R2: It’s good!

R1: And we used to laugh ab- about it that ‘eii you this boy-wo. The day your husband catches you… oh, you’ll never know’. Focus group (24–49-years old)

This type of secret use of contraception was a well-known tactic among respondents, particularly secret use of injectables:

Most of the cards for family planning [i.e. record cards] are in the […] facilities. They don’t take them home. Especially those who go for the Provera [3-month injectable]. So when they come there they mention their number, or something they have their numbers on their phones and things. They just mention, look through, get it, and then give them the injections. They don’t take it home at all. So there is no way their husband will ever get to know. Focus group (25–49-years old)

Women acting individually and using silence and secrecy was a response to lack of dialogue and agreement between partners regarding desired number and timing of children, but it was also talked about in terms of socio-cultural and religious norms. Discomfort about referring to sex even obliquely, as well as religious prohibitions on contraceptive use and premarital sex also helps maintain silence around fertility control.

### Being fertile and protecting menses: ‘that thing is supposed to come out’

Being able to become pregnant when they choose appears in the women’s narratives as an aspect of their bodily autonomy and also more broadly as a part of control over one’s life which involves having to combine common and apparently opposing subject positions to meet career, education and childbearing expectations. Participants had delayed or were delaying first pregnancy to complete various levels of education and talked about education as ‘wasting’ one’s fertile life:

R: I think that, em, when somebody is too educated before marriage, it means that, you delay if you want to really go high in the education before getting married you delay and so part of your fertility period is already gone…

R: Wasted.

R: … and so the rest that is remaining […]

R: … yes. The, the… the rest that is remaining you, you don’t want to take chances. That is how come some will never go for hormonals […]. Focus group (25–49-year olds)

One participant, K, told us about some of the stories she had heard about hormonal method side effects. She says it is important to educate young women on how to manage their menses (i.e. menstrual bleed). She connects ‘stopping’ the flow of menstrual blood with becoming infertile and draws on normative ideas regarding common conceptions of menses (e.g. as a way the body becomes clean) and how menses should be managed, including the importance of letting blood flow. Implicit in this account is a view of menses as a signifier of fertility:

When you are bleeding, how can you have sex? But these girls of today, they say they take milk to clean their under [vulva/vagina] before they just go and do it but I think it is not the best because the dirt is supposed to come out so I don’t know why they have to go and push it back inside again. […] They will put it [milk or beer] in cotton and then put it, they will insert it […] I told them that is not the best because that thing [menstrual blood] is supposed to come out. That is the dirt in you. […] How they stop it [by having sex], it will create problems for them. It can even block their wombs. It can give them fibroid. It can even kill them because you don’t know, while you are pushing this thing, you don’t know the infection that it is going to give you. And in future if you don’t give birth […] I think you should go and tell them […] the importance of the blood flowing.

Being confident that they can become pregnant rapidly and evidence of ‘normal menses’ help women feel they can comply with the ‘gender contract’ and potentially avoid infidelity or divorce, which are common concerns in relation to inability to have children (Fledderjohann, 2012). Infertility, for instance, it was widely agreed, would lead to a woman’s marriage failing.

Women’s narratives about the need for pregnancy on demand included accounts of dialogue with others (family members and husbands) who make women accountable for their ‘gender contract’:

When you get married there is a pressure on you to have kids, you know so everybody is waiting. ‘What’s happening?’ You know? Interviewee R (38-years old)

Participants’ concerns about the biomedical effects of hormonal methods (often referred to as ‘drugs’ or ‘artificial’ methods) were often articulated as concerns about effects on menses. Participants shared normative ideas about how menses should be, such as the number of days that bleeds should last and quantity of blood that should flow. Their narratives suggest that these normative ideas and aspirations mediated women’s attitudes to modern hormonal methods and their fertility regulation practices:

I did the one-month injection. I did the two-month. It didn’t work for me. If I say it didn’t work for me, I mean I realise my menses was not really flowing like how it is supposed to flow. I tried the two; I didn’t really see anything. Like, that one too, my menses wasn’t really flowing well. And I tried the, I tried the three-month too, and then for the, with the one under the arm [implant], it [my menses] seized [stopped] for a whole year. My menses didn’t come for a whole year and I, I didn’t really feel comfortable about that […] my menses wasn’t flowing ‘cause I thought it was piling up under my abdomen. That is why I went to remove it [the implant]. Interviewee N (37-years old)

Biomedical concerns, fears of infertility, delayed pregnancies, infidelity and marriage break up are instigated by others and produced socially through rumours circulating within participants’ social environment, helping shape women’s attitudes and decisions about contraception and abortion. An example of how this dynamic can work emerged in the age 25–49-year focus group. One participant referred to abstinence as a ‘holiday period’, and the others laughed. The woman explained that she did not like hormonal methods and so preferred abstinence. Another woman in the group then made the participants laugh even more by asking the first woman what was she doing to satisfy her husband, reproducing the same social pressures Ghanaian women face from extended family members; the need to be ‘a good wife by being sexually available to your spouse’ (Sossou, 2007). She asked the same question several times during the discussion and implied that when men do not have sex within marriage, they seek it elsewhere.

Prioritising ‘protecting’ menses meant that women considered calendar methods the best way to regulate fertility, sometimes in combination with ECPs, withdrawal and/or condoms. Their use of calendar methods relied on the idea that certain times of the menstrual cycle were completely ‘safe’, that is, carried no risk of pregnancy. The additional methods were typically used during the other, ‘unsafe’ times, as opposed to during the ‘safe’ period which many women implied in their accounts they believed to carry no risk of pregnancy at all and hence no need for additional measures to reduce pregnancy risk.

### Combining methods to be in control, yet feeling uncertain: ‘I see my monthly cycle: Oh! I thank God’

Using numerous contraceptive methods together, women make choices and take action, but are also responding to the uncertainties arising from their need to conform to their male partner’s preferences and using comparatively unreliable withdrawal and calendar methods. Participant R, for instance, introduces her husband in her account about personal views on condoms and articulates her own preferences through and against her husband’s:

I dislike [withdrawal] … There is the tendency that you might make a mistake [respondent laughs]. A slight mistake, you know, so I am particular about that. That is the reason why, um, if I’m safe [i.e. if she perceives herself to be in the ‘infertile’ days of her menstrual cycle] fine – then I could do the withdrawal and then I know. But if I am not too sure about my timing, then I’ll, I will force for him to use the condom. […] I think my husband, even though he is okay with it, wouldn’t want to put it [the condom] on early. He wouldn’t want to wear it earlier; he will wait for some time before he would wear it. I don’t have a problem with it anyway […] You know I mean the condom can do all the job so why put yourself through any more stress. […] So, umm, I mean, once I knew I had the condom I didn’t have any problem with thinking of calculating, or… you know? […] All the things that I didn’t want, the condom will spare me that, not having… So I don’t have to have kids when I don’t want it. I can still have my fun, you know, still have sex and be okay […] you know, having to… whilst having sex you still have to think about the withdrawal and all those things. […] It [the condom] would take care of everything. Interviewee R (38-years old)

She portrays condoms as ‘most reliable methods’ to avoid the uncertainty and ‘stress’ which comes from using other methods (withdrawal). Although she says her husband will be okay with the condoms, she still needs to ‘force’ him to use one and he will delay putting it on. This interviewee had already had children and so may feel she has met her social responsibilities in this regard, which in turn may help her to ‘force’ condom use rather than relying on tactics requiring secrecy or silence.

Participants emphasised that they could not rely on men to withdraw in time and talked about withdrawal as something largely outside their control. Because of this, the women took responsibility for calculating the ‘safe’ period. Some said they worried that they might miscalculate. Unsurprisingly, using low-efficacy (calendar and withdrawal) methods led to unplanned pregnancies and abortions.

There was a sense that women expected men to be reluctant to use condoms but that women could mitigate the resulting risks of pregnancy by taking ECPs. For instance, one focus group participant invokes the voice of male partners (‘you are promiscuous’) to talk about how women are positioned by others if they mention condom use. ECPs also help avoid these social risks:

Well I don’t think, when it, when, when you come to the Ghanaian setting you know women don’t really decide whether the partner should use condom or not. So, um, using condom is not always during sex but, woman just don’t decide, […] they have no say in whether you should use condom or not. You know when a woman raises the issues of condoms they have this, ‘you are promiscuous’ or something that comes in mind. So a woman who don’t even, er… who knows, um… she is not safe for the, for the month and then has unprotected sex, will use a pill [i.e. ECP]. Focus group (18–24-year olds)

The contrast between taking control yet also feeling uncertain is contained in many participants’ narratives about the tensions between different subject positions (women meeting reproductive needs and women conforming to patriarchal subject positions).

Participant V, for example, positions herself as making choices over different sex and contraception ‘styles’ and presents herself as having fun taking risks and having agency over contraceptive decision making. Yet, she also talks about the concerns she experiences when using withdrawal and mentions negative ‘afterthoughts’ and the need for ECPs as a safeguard:

I don’t know about other women, but I start it [having sex] at dawn. It’s cooler that way [both laugh], it cooler that way so at dawn you go for the raw meat, and then maybe later in the day after breakfast lunch you want to go for the condom, and then in the evening you want, you just want it all in, and afterwards you go for the EC pill. […] You can try all styles you want [both laughing] without being bothered. [laughs] Yeah and then the disadvantage one is, um, the skin to skin sometimes you start thinking ‘ey there’s a little bit stuck in me’, like it’s not a fun thing, the afterthoughts… the afterthoughts… but apart from that you enjoy everything else. […] It’s fun that way besides it’s… Apart from the fact that you have, you will be insecure after the sexual intercourse, maybe, especially if you are skin to skin and… or for the withdrawal method you will be a bit insecure till you see your next period you… It’s fun it’s like you taken some risk and then… […] I really have a lot of sex and then um I see my monthly cycle: Oh! I thank God! I get happy! [laughter] I get so excited I don’t even think about the pain that it will bring but just the fact that it came! Oh! It’s just good news! Interviewee V (22-years old)

ECPs hold the promise of pregnancy prevention but can also foster anxiety if they cause a later bleed:

[ECPs] can even make you panic if you take, like, maybe more than two or three in a month, your period might delay and you think you are pregnant and all that. Interviewee X (23-years old)

## Discussion

We have shown how fertility regulation practices are both shaped by and are responsive to socio-cultural norms and express and maintain women’s social identities. Women’s accounts of fertility regulation practices contain plural discourses about identity (i.e. who a woman is, how she should be and how she should behave) and reflect women’s efforts to negotiate the hybrid subject positions they occupy.

Our participants’ identities were anchored in competing subject positions for these educated women, where ‘modern’ and ‘traditional’ social expectations co-exist. Contraceptive practices were socially negotiated and responsive to women’s liminal social position: both with and without agency, both with strong professional career aspirations and with desire to comply with traditional norms of motherhood. By acting secretly, women sustained a hybrid identity that combined modern and traditional subject positions around womanhood in terms of their obligations to bear children, while also meeting modern educational and employment responsibilities and aspirations.

In other settings (Basu, 2005), a decline of use of ‘modern’ contraception among educated women has been interpreted as representing individual acts of control over one’s body, postmodern choices of women defying medicalisation in favour of ‘natural’ or ‘traditional’ fertility regulation. A trend towards the ‘organic’ and ‘natural’ may help explain some of the aversion to highly effective contraceptive methods (which are seen as ‘unnatural’), and certainly may be invoked in explanations about choices to use ‘natural’ methods (although this was not the case in our study). Far more important in this study, however, was not women’s individual fertility preferences – inasmuch as these can be said to exist separately from society at all – but their limited agency and the social pressures about ‘risk’ in terms of fertility and in terms of relationships. It seems likely that similar concerns will be salient in other settings too.

Participants often described their strong commitment to their education and to developing successful careers in paid work – we only interviewed women who had already completed a minimum of secondary education. However, some also described the education period required by the modern society they live in as a ‘waste’ of their fertile lives, or as holding them back, in terms of leaving less time to fulfil their felt obligation to procreate. Educated women may particularly occupy and negotiate multiple and often seemingly competing positions: achieving a good level of education and also being responsive to husbands’ desires (‘being a good wife’ (Sossou, 2007)), protecting their marriage and family in responding to the high value placed on women’s fertility, while also attempting to control the timing, spacing and number of their births. Our work extends previous studies by showing how women navigate these constraints using different tactics to negotiate and attempt to control their reproductive futures. It also contributes to previous health psychology work (Campbell and Murray, 2004; Jovchelovitch and Gervais, 1999) by illustrating the psycho-social processes through which health beliefs (in our case about contraception) and related practices are intertwined with constant identity maintenance and negotiation.

Our participants seemed to place a great deal of faith in the ‘safe’ period being truly ‘safe’ from pregnancy risk and so typically used no additional contraceptive method in the ‘safe’ period – a method well known for being prone to failure (Che et al., 2004). If women are convinced, the ‘safe’ period is fully safe (providing they calculated it correctly), then they do not need to worry about their partner disapproving or fear loss of fertility. For this reason, they may simply prefer to endure the ‘control/uncertainty’ condition that many women described because this helps them avoid social disadvantages. Secret use of more effective contraceptives, while reducing women’s uncertainty about pregnancy, also carries its own disadvantages and uncertainties such as fear of being found out or fear of loss of fertility.

This study shows how fertility regulation choices and practices are fundamentally dialogical and become an important way to maintain identity – and this is a feature of fertility regulation that is also likely to be relevant elsewhere.

Investigating and illuminating the dialogical nature of fertility aspirations, social identity and socio-cultural norms helps us to understand how and why women regulate their fertility. Better supply of contraceptive pills, for instance, may not increase use without corresponding education to service providers and women about the effects on fertility and menstruation. Failing to recognise that women may need to use methods secretly may also reduce the usefulness of services.

While women’s individual choices and capacities to meet their reproductive needs is often the focus of mainstream reproductive rights work (Silliman et al., 2002), few attempts have been made to understand or address the complex tactics women must use to preserve and develop social identities, which may well determine what tactics they will use or be prepared to consider for regulating fertility. This may particularly be the case for educated women who might be assumed to ‘know’ enough and have good enough access to contraception that they are not seen as needing any specific intervention. Knowledge may be necessary but is not sufficient to change contraceptive behaviour, even when women intend to use contraceptives (Kiene et al., 2014). In Ghana, as urbanisation and women’s educational aspirations increase, the tensions between subject positions felt by our participants – educated, urban women from the capital – may well be felt by wider groups of women, and programmes need to try to address these issues with information, promotion and commodities provision that takes these tensions into account. Programmes should create ‘safe spaces’ (Campbell and Jovchelovitch, 2000; Campbell and Murray, 2004; Priego-Hernandez, 2015) for women to reflect on how to control their fertility needs in a way that responds to their personal aspirations and projects while allowing them to protect their social position. Such programmes, taking a participatory approach, could help stimulate critical reflective dialogue and renegotiation of the social identities that constrain women’s agency and control over their fertility (Campbell and Jovchelovitch, 2000; Campbell and Murray, 2004). Programmes must also address women’s wider social contexts (Campbell and Cornish, 2010; Campbell and Murray, 2004; Vaughan, 2010) and aim to develop ‘enabling environments’, that is, that help women to take control over their fertility. Simply informing women that their preferred calendar method is ineffective and supplying hormonal methods instead seems unlikely on its own to change practice given the complex social features of contraceptive use we identify here.

Family planning programmes that fail to take social dimensions into account risk maintaining the reproductive status quo of the women they aim to help, reproducing social spaces where women still struggle both to claim their reproductive rights and to constitute themselves as subjects of those rights.
